# Goal-directed fluid therapy using uncalibrated pulse contour analysis and balanced crystalloid solutions during hip revision arthroplasty: a quality implementation project

**DOI:** 10.1186/s13018-023-03738-0

**Published:** 2023-04-06

**Authors:** R. F. Trauzeddel, M. Leitner, L. Dehé, M. Nordine, S. K. Piper, M. Habicher, M. Sander, C. Perka, S. Treskatsch

**Affiliations:** 1 Department of Anesthesiology and Intensive Care Medicine, Charité - Universitätsmedizin Berlin, Corporate Member of Freie Universität and Humboldt Universität zu Berlin, Charité Campus Benjamin Franklin, Hindenburgdamm 30, 12203 Berlin, Germany; 2 Institute of Medical Informatics, Charité –Universitätsmedizin Berlin, Corporate Member of Freie Universität Berlin and Humboldt-Universität zu Berlin, Charitéplatz 1, 10117 Berlin, Germany; 3 Institute of Biometry and Clinical Epidemiology, Charité –Universitätsmedizin Berlin, Corporate Member of Freie Universität Berlin and Humboldt-Universität zu Berlin, Charitéplatz 1, 10117 Berlin, Germany; 4 Department of Anesthesiology, Operative Intensive Care Medicine and Pain Therapy, Justus Liebig University of Giessen, Rudolf-Buchheim-Straße 7, 35392 Gießen, Germany; 5 Center for Musculoskeletal Surgery, Charité –Universitätsmedizin Berlin, Corporate Member of Freie Universität Berlin and Humboldt-Universität zu Berlin, Campus Charité Mitte and Virchow-Klinikum, Charitéplatz 1, 10117 Berlin, Germany

**Keywords:** Goal-directed fluid therapy, Perioperative, Uncalibrated pulse contour analysis, Balanced crystalloid solutions, Balanced colloid solutions, Hip revision arthroplasty

## Abstract

**Background:**

To implement a goal-directed fluid therapy (GDFT) protocol using crystalloids in hip revision arthroplasty surgery within a quality management project at a tertiary hospital using a monocentric, prospective observational study.

**Methods:**

Adult patients scheduled for elective hip revision arthroplasty surgery were screened for inclusion in this prospective study. Intraoperatively stroke volume (SV) was optimized within a previously published protocol using uncalibrated pulse contour analysis and balanced crystalloids. Quality of perioperative GDFT was assessed by protocol adherence, SV increase as well as the rate of perioperative complications. Findings were then compared to two different historical groups of a former trial: one receiving GDFT with colloids (prospective colloid group) and one standard fluid therapy (retrospective control group) throughout surgery. Statistical analysis constitutes exploratory data analyses and results are expressed as median with 25th and 75th percentiles, absolute and relative frequencies, and complication rates are further given with 95% confidence intervals for proportions using the normal approximation without continuity correction.

**Results:**

Sixty-six patients underwent GDFT using balanced crystalloids and were compared to 130 patients with GDFT using balanced colloids and 130 controls without GDFT fluid resuscitation. There was a comparable increase in SV (crystalloids: 65 (54–74 ml; colloids: 67.5 (60–75.25 ml) and total volume infused (crystalloids: 2575 (2000–4210) ml; colloids: 2435 (1760–3480) ml; and controls: 2210 (1658–3000) ml). Overall perioperative complications rates were similar (42.4% (95%CI 30.3–55.2%) for crystalloids and 49.2% (95%CI 40.4–58.1%) for colloids and lower compared to controls: 66.9% (95%CI 58.1–74.9)). Interestingly, a reduced number of hemorrhagic complications was observed within crystalloids: 30% (95%CI 19.6–42.9); colloids: 43% (95%CI 34.4–52.0); and controls: 62% (95%CI 52.6–69.9). There were no differences in the rate of admission to the post-anesthesia care unit or intensive care unit as well as the length of stay.

**Conclusions:**

Perioperative fluid management using a GDFT protocol with crystalloids in hip revision arthroplasty surgery was successfully implemented in daily clinical routine. Perioperative complications rates were reduced compared to a previous management without GDFT and comparable when using colloids.

*Trial registration*: ClinicalTrials.gov identifier: NCT01753050.

## Background

Hip revision arthroplasty is a frequently performed surgical procedure and is associated with perioperative complications such as myocardial injury, postoperative cognitive dysfunction and delirium, prolonging intensive care and hospital length of stay (LOS) [1–5]. In this context, the application of goal-directed fluid therapy (GDFT) has been discussed as a potential intra-operative counter-measure against these aforementioned complications [6]. Its goal is to ensure and optimize organ perfusion by improvement of oxygen delivery and supply in combination with advanced hemodynamic monitoring [7].

The concept of GDFT has been applied to various surgical and intensive care scenarios in the last decade [8–11]. The aim of the previously published HIPHOP I study was to improve perioperative outcomes of patients undergoing hip revision arthroplasty. It was performed as an ambispective interventional study and was part of a quality management project accompanying the implementation of a GDFT protocol compared to patients without any kind of GDFT or special volume measurement. Stroke volume (SV) was measured with an uncalibrated pulse contour analysis and was optimized according to a standardized treatment protocol using balanced colloid solutions. The results showed a significant reduction in intensive care and hospital LOS, perioperative bleeding rates and overall complications when GDFT was applied compared to standard care [12].

However, over the last years, ambiguous results have been reported regarding the perioperative use and safety of colloid solutions. Prior studies have suggested that intra-operative colloid use is associated with a higher rate of postoperative renal failure, need for renal replacement therapy and coagulation disorders [13–17], whereas other studies did not find significant outcome differences when compared to crystalloid solutions [18, 19]. Due to conflicting evidence, the European Medicines Agency (EMA) has finally issued a suspension of intra-operative colloid usage. In the following, previous studies have shown that using a GDFT protocol with balanced crystalloids for patients undergoing major abdominal or cardiothoracic surgery or on intensive care unit (ICU) is in fact feasible [20–22]. Similar effects might be thus expected in major orthopedic surgery. In this context, the aim of this study was the implementation of a GDFT protocol using balanced crystalloids in hip revision surgery in daily clinical routine as part of a quality management project. Quality of perioperative treatment was assessed by protocol adherence, SV increase as well as the rate of perioperative complications. Its findings were then compared to the previously published HIPHOP I trial.

## Methods

### Ethics

All procedures involving humans were in accordance with the ethical standards of the institutional research committee and with the 1964 Declaration of Helsinki and its later amendments. The original study as well as the amendment was approved by the local ethics committee at Charité–Universitätsmedizin Berlin (EA 1/315/12, Chairperson Dr. R. Uebelhack) (date of approval of the original study 05/12/2012, date of approval of the amendment 04/10/2014) and registered at ClinicalTrials.gov on 20/12/2012 (NCT01753050). Informed written consent was obtained from all study patients in the respective prospective parts.

### Study design

This study was designed as a prospective study and amended upon a previous ambispective study performed at the Charité–Universitätsmedizin Berlin (HIPHOP I) [12]. The study was performed at the Charité–Universitätsmedizin Berlin, Berlin, Germany. Stroke volume monitors were provided by Edwards Lifesciences, which had no influence in the study design and data analysis.

### Study population

**Fig. 1:**
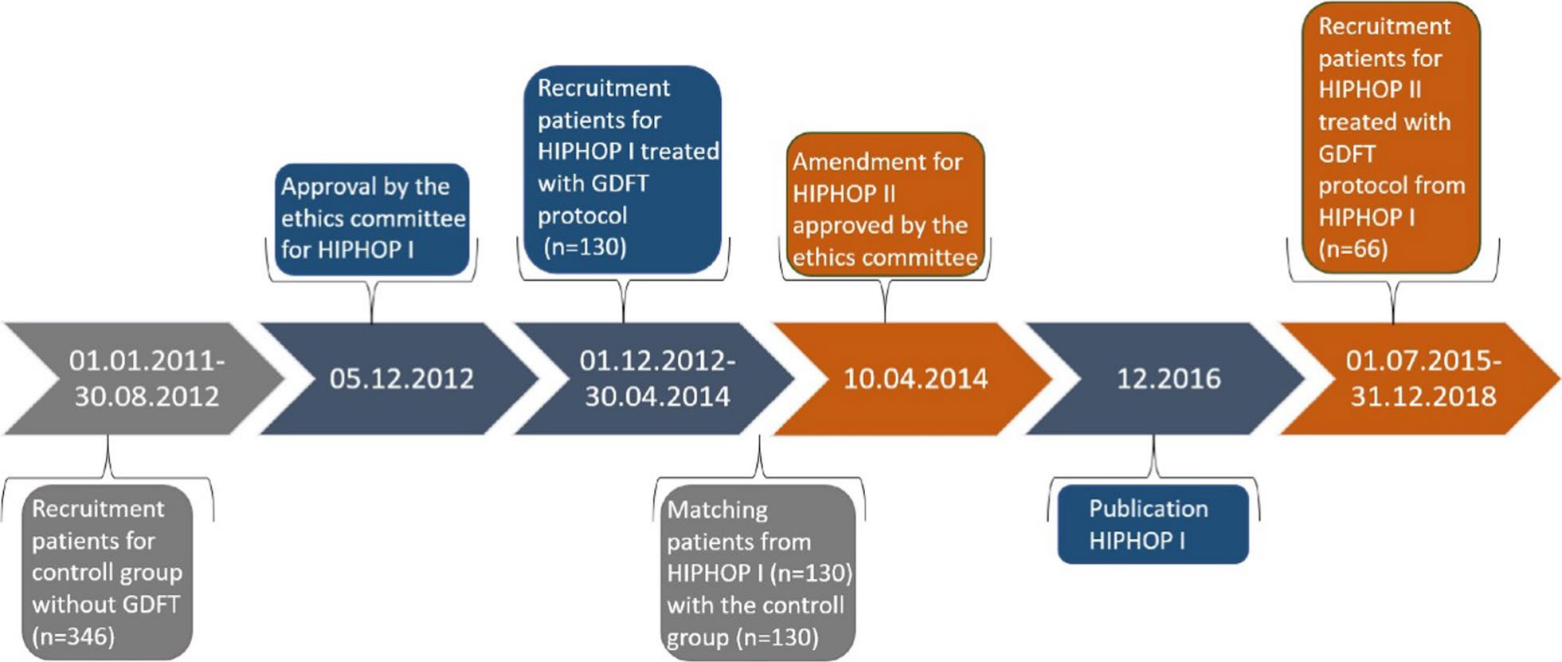
Timeline recruitment periods. Gray: control group, blue: colloid group, orange: crystalloid group

All patients admitted for elective revision hip surgery were screened for study inclusion. Inclusion criteria were age ≥ 18 years and one of the following surgical procedures: hip revision with change of the prosthesis, removal of existing hip arthroplasty or new implantation of hip prosthesis in patients after Girdlestone resection arthroplasty (“hip revision surgery”) including aseptic as well as septic pathophysiologies. Exclusion criteria were as follows: pregnancy, patients who were not able to consent to study participation, atrial fibrillation or other severe heart rhythm disorders which impeded usage of uncalibrated pulse contour analysis or previous participation in the HIPHOP I study or in another clinical intervention trial with a similar study objective. Patients of the crystalloid group were compared with a previous study consisting of two different historical cohorts: (a) patients managed intraoperatively according to the discretion of the attending anesthesiologist before implementation of a GDFT protocol (retrospective control group) and (b) patients prospectively managed according to the below described GDFT protocol using colloid bolus applications (prospective colloid group) [12]. The recruitment periods for the control group, the colloid group and the crystalloid group are shown in Fig. 1.

### Study protocol

Study participants in the colloid group as well as in the crystalloid group were intraoperatively managed according to our GDFT protocol as previously described in the literature [12]. All groups received a standard infusion of crystalloids as basal maintenance rate. All patients underwent general anesthesia, which was performed according to our local standard operating procedure (SOP) with Fentanyl (1–2 µg kg^−1^), Propofol (1–2 mg kg^−1^) and Cisatracurium (0.15 mg kg^−1^) as this was the standard anesthetic procedure at our institution. Anesthesia was maintained to the discretion of the attending anesthesiologist with Sevoflurane or Propofol as well as intermittent boli of Fentanyl and Cisatracurium as needed. Instead of balanced colloid solutions (Volulyte® 6%, Fresenius Kabi Deutschland GmbH, Bad Homburg, Germany, or Gelafundin ISO 40 mg ml^−1^, B. Braun Melsungen AG, Melsungen, Germany) as used in the former study (colloid group) [12], balanced crystalloids (Sterofundin® ISO, B. Braun Melsungen AG, Melsungen, Germany) were used in the current study (crystalloid group) within the same optimization protocol. After establishing standard monitoring, including invasive blood pressure measurement via right or left radial artery, SV analysis was monitored using an uncalibrated pulse contour analysis (EV1000, Edwards Lifesciences, Irvine, CA, USA) and a special pressure transducer (FloTrac system, Edwards Lifesciences). After determining individual baseline SV (SV_baseline_), an intravenous bolus of 250 ml of balanced crystalloid fluid replacement solution was given within 5 min and consecutively repeated until no further increase of SV ≥ 10% could be achieved. The last successful fluid challenge resulting in an SV increase > 10% defined the optimum SV (SV_opt_). According to the study algorithm, only a single type of crystalloid in the actual or colloid in the previous study was given. SV indicating the need for further fluid replacement in the ongoing course of the operation was named SV_trigger_ and was defined as SV_opt_ minus 10%. During surgery, the hemodynamic goal was to maintain SV above SV_trigger_ using aforementioned fluid boli. If SV_trigger_ was not undershot, but the patient showed signs of hypoperfusion, additional fluid or vasoconstrictive drugs could be administered based on clinical judgment. In case of unresponsiveness of SV to fluid boli, inotropic therapy (e.g., PDE-III-inhibitor or beta-adrenergic drug) could also be initiated at the discretion of the attending anesthesiologist in the absence of ≥ 2 contraindications: insulin-dependent diabetes mellitus, impaired renal function, existing coronary heart disease or angina pectoris, heart failure (NYHA classification) and previous stroke. Volume optimization was only performed with fluids and inotropes, and vasopressors were given *add on* only in case of arterial hypotension independent of SV readings to achieve a mean arterial pressure (MAP) ≥ 65 mmHg. The used inotropic drug (PDE-III-inhibitor or beta-adrenergic drug) was based on an individual decision of the attending anesthesiologist, depending on the patient’s situation, preexisting diseases and contraindications for every drug. Furthermore, the protocol allowed the attending anesthesiologist to administer colloid solutions, blood products, or to start an additional cardiovascular therapy based on clinical judgment at any time. However, tranexamic acid (TXA) was applied intravenously to all patients according to the local SOP.

Defined protocol deviations in our study were the following: inotropes given despite undershooting of SV_trigger_ based on clinical judgment, inotropes not administered despite SV_trigger_ indication as defined above, length of surgery below 90 min in total or choosing another dosage of inotrope support as defined in the GDFT protocol.

### Outcome variables

The increase in SV and the total amount of volume given in the GDFT crystalloid group was compared to the GDFT protocol with colloid solutions. The following predefined perioperative complications were tracked during the entire hospital stay where surgery took place: infectious complications (wound infections, wound healing disturbances, pneumonia, urinary tract infections, sepsis, endocarditis and peritonitis), cardiac complications (arrhythmias requiring medical treatment, pulmonary edema and embolism, myocardial infarction, cardiac arrest), neurological complications (postoperative delirium and stroke), renal complications (increase of creatinine above twofold before surgery or need for dialysis) and hemorrhagic complications (postoperative bleeding with the need for red blood cell (RBC) transfusion) [12], total length of postoperative hospital stay, rate of admission to the post-anesthesia care unit (PACU) or ICU and overall proportion of patients developing one or more postoperative complications during hospital stay. Intraoperative blood loss was estimated by the total amount of blood in the surgical suction cup at the end of the operation (without flushing liquids) in accordance with the surgeon’s validation of this amount.

### Statistical analysis

Statistical analysis was done using SPSS® Statistics 25 (SPSS, Inc., Chicago, IL). Due to the sample size non-symmetrical distribution was assumed. Results are expressed as median with 25th and 75th percentiles for quantitative data or absolute and relative frequencies for categorical data. Complication rates are further given with 95% confidence intervals for proportions using the normal approximation without continuity correction. To explore statistical differences, quantitative data were compared using the Kruskal–Wallis test or Mann–Whitney *U* test and frequency data using chi-square test. A *p* value < 0.05 was considered statistically significant without adjustment for multiple comparison. All *p* values constitute exploratory data analyses and do not allow for confirmatory generalization of results.

To assess the adherence to the GDFT protocol, the total number of interventions and the number of protocol deviations of every patient were counted. They are shown as absolute and relative terms with interquartile ranges where appropriate.

## Results

**Fig. 2:**
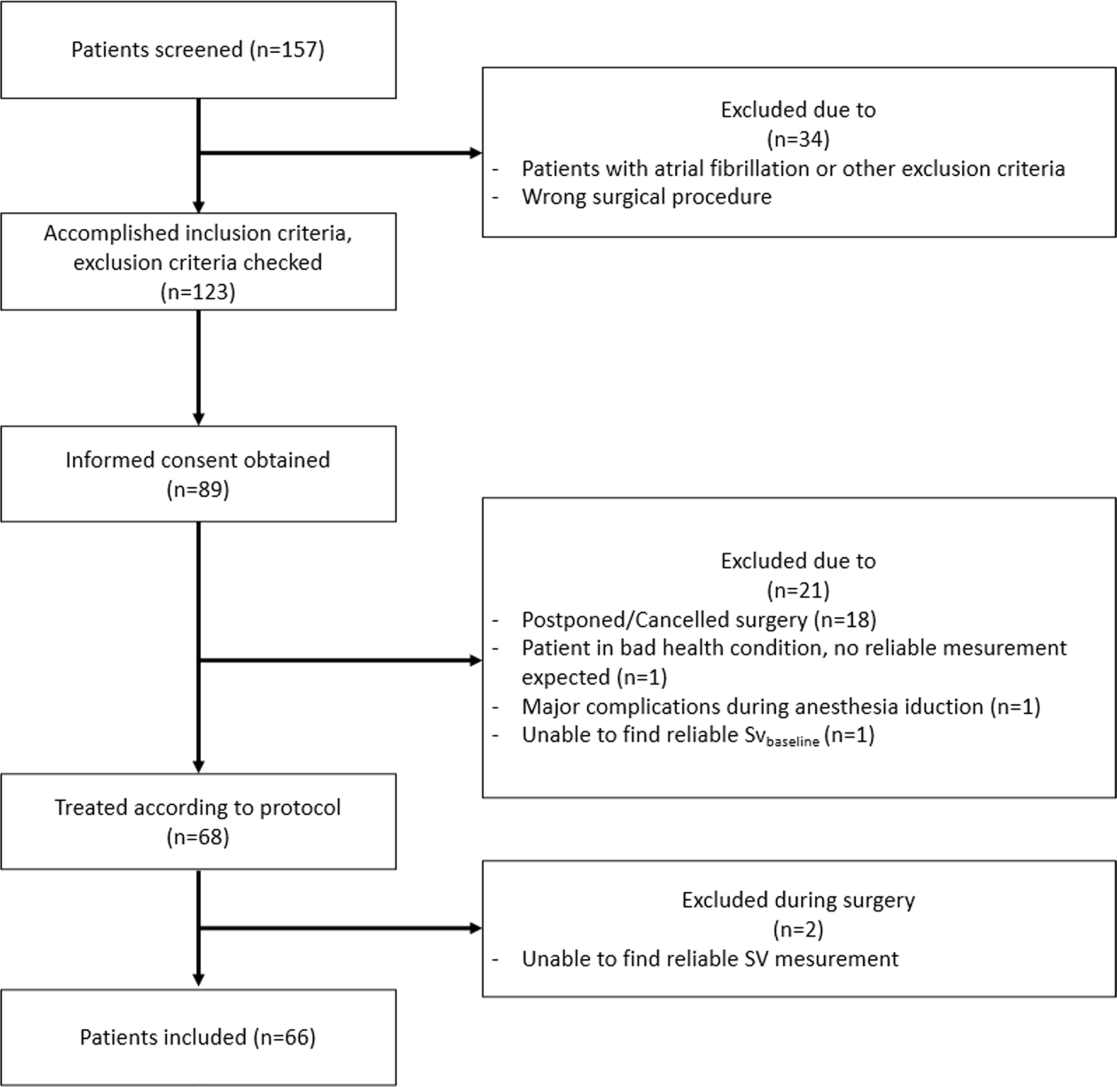
Patients enrollment—ITT

Between July 2015 and December 2018, 157 operative patients were screened. Sixty-six of them could be included for data analysis. The enrollment is shown in Fig. 2. Baseline characteristics were similar between groups (Table 1).

**Table 1:** Baseline characteristics of the study population (ITT analysis)

	Crystalloid group (n = 66)	Colloid group (n = 130)	Control group (n = 130)
Age (years)	68 (55–77)	71 (62–75)	72 (60–76)
Female (n, %)	41 (62%)	81 (62%)	86 (66%)
Body height (cm)	169 (161–178)	168 (163–175)	166 (160–171)
Body weight (kg)	79 (68–90)	79 (64–90)	76 (65–85)
BMI (kg/m^2^)	27.6 (24.4–31.1)	27.8 (23.8–32.1)	27.4 (24.7–30.1)
CCS [44]	2 (1–4)	3 (2–4)	3 (2–5)
ASA score	2 (2–3)	2 (2–3)	2 (2–3)

Median with 25th and 75th percentile or absolute and relative frequencies (indicated by a percentage sign)

*BMI* Body mass index,* CCS* Charlson Comorbidity Score,* ASA* American Society of Anesthesiology

In total, including the colloid and the crystalloid group, 2066 study interventions were performed with 219 protocol deviations (10.6%). The study protocol was followed in 98.5% of all cases (median: 100% (IQR 100–100)) and in the crystalloid group with 589 study interventions including 9 (1.5%) protocol deviations. The study protocol in the colloid group was followed to 88% (median: 100% (IQR 83.3–100)) comprising of 1477 study interventions with 170 (12%) deviations to protocol. In 57 out of 66 patients (86.4%) in the crystalloid group and in 66 out of 130 patients (50.8%) in the colloid group, the protocol was followed to 100%. The following individual protocol deviations were noted: (a) 1 patient received inotropes due to clinical assessment although SV_trigger_ was not undershot, (b) 1 patient received no inotropes even though SV_trigger_ was undershot but MAP was high, (c) 3 patients received inotropes in the wrong dosages, and (d) 4 patients with SV under SV_trigger_ did not receive inotropes as surgery was close to ending.

Using crystalloids, a comparable increase in SV was achieved compared to the GDFT colloid group and cumulative amount of infused volume within the GDFT protocol did not differ between both groups (crystalloid group: 2575 ml, colloid group: 2435 ml, control group: 2210 ml). Intraoperative data of all groups are shown in Table 2. Twenty-five patients (37.9%) in the crystalloid group and 20 patients (25.4%) in the colloid group were treated with vasopressors. In the crystalloid group, 19 patients (28.8%) were treated by inotropes. Eleven out of these 19 patients received dobutamine, and 8 patients were treated with enoximone. Ten patients with the need for inotropic therapy also got norepinephrine. In the colloid group, 28 patients (21.5%) were treated with inotropes, whereas just 7 of these also were in need for norepinephrine. According to the protocol, patients in the control group received less inotropic therapy (18 out of 130) (13.8%) than patients in the colloid and crystalloid group (*p* < 0,001). Prolonged postoperative need to infuse norepinephrine among all three groups did not differ. Duration of anesthesia and surgery was comparable in all groups.

**Table 2:** Intraoperative data (ITT analysis)

	Crystalloid group (n = 66)	Colloid group (n = 130)	Control group (n = 130)	p
Anesthesia time (min)	188 (145–250)	197 (170–254)	185 (160–230)	0.168^a^
Surgery time (min)	119 (84–162)	135 (107–171)	125 (99–159)	0.063^a^
Cumulative amount of infused volume (ml)	2575 (2000–4210)	2435 (1760–3480)	2210 (1658–3000)	0.063^a^
Crystalloids (ml)	2250 (1750–3000)	725 (500–1000)	1500 (1000–2000)	< 0.001^a^
Colloids (ml)	0 (0–0)	1250 (1000–1750)	500 (500–1000)	< 0.001^a^
Median SV (ml)	65 (54–74)	67.5 (60–75.25)	-	0.96^c^
Inotropes (%)	19 (28.8%)	28 (21.5%)	18 (13.8%)	< 0.001^b^
Blood transfusion (%)	18 (27.3%)	57 (43.8%)	47 (36.2%)	0.071^b^
Intraoperative blood loss (ml)	800 (475–1200)	1000 (500–1500)	800 (485–1500)	0.562^a^
Necessity NE	25 (37.9%)	20 (25.4%)	31 (40.3%)	0.002
Average max. NE-dose (µg/kg Bodyweight/min)	0.027	0.0133	0.0191	0.004^a^
NE at end of surgery	10 (15.2%)	10 (7.7%)	18 (13.8%)	0.185^b^
Postoperative admission to				0.223^b^
Recovery room	45 (68.2%)	71 (54.6%)	75 (57.7%)	
PACU	18 (27.3%)	53 (40.8%)	44 (33.8%)	
ICU	3 (4.5%)	6 (4.6%)	11 (8.5%)	
LOS				
Recovery room (min)	150 (115.5–190)	160 (110–205)	170 (120–240)	0.177^a^
PACU/ICU (min)	750 (427.5–1235)	400 (207–825)	960 (360–1210)	< 0.001^a^

Parameters are shown as median with 25th and 75th percentile or absolute and relative frequencies (indicated by a percentage sign)

*NE* Norepinephrine,* PACU* Post-anesthesia care unit

All *p* values constitute exploratory analysis comparing overall differences between all three groups: ^a^Kruskal–Wallis test, ^b^chi-square test, ^C^Mann–Whitney *U* test

**Fig. 3:**
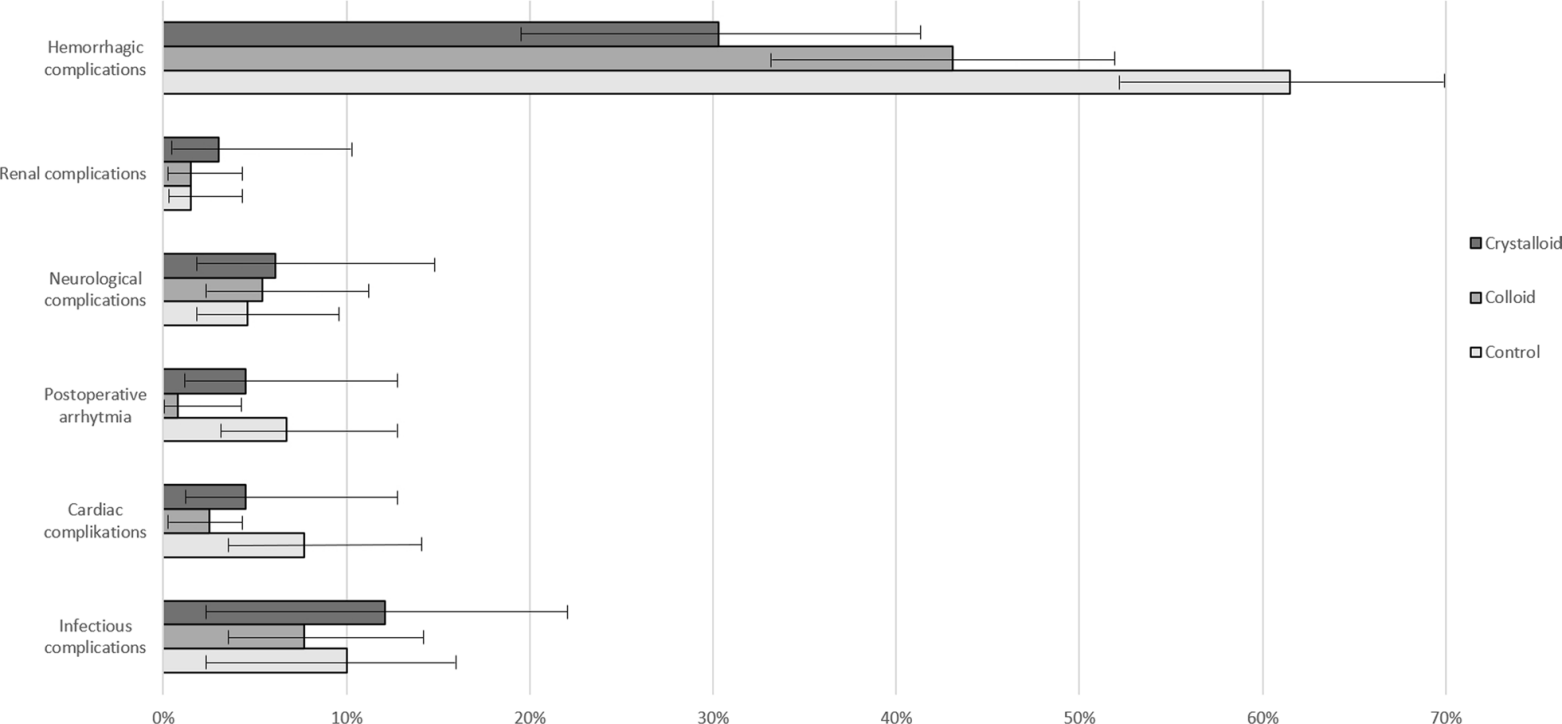
Complication rates between the three groups

Overall, across all patients in the intervention groups, postoperative complication rates were similar to 42% (*n* = 28) (95%CI 30.3–55.2) for the crystalloid and 49% (*n* = 64) (95%CI 40.4–58.1) for the colloid GDFT group, and significantly lower compared to control patients without GDFT (67% (*n* = 87) (95%CI 58.1–74.9)) (Fig. 3). This pattern was mainly characterized by hemorrhagic complications, which were considerably lower in the GDFT crystalloid group (30% (*n* = 20) (95%CI 19.6–42.9)), compared to the GDFT colloid group (43% (*n* = 56) (95%CI 34.4–52.0)) and the control group (62% (*n* = 80) (95%CI 52.6–69.9)), respectively. There was as similar need for blood transfusions intraoperatively without any events of massive transfusion. The rates for postoperative arrhythmia, neurological, renal, cardiac and infectious complications were not significantly different.

**Fig. 4:**
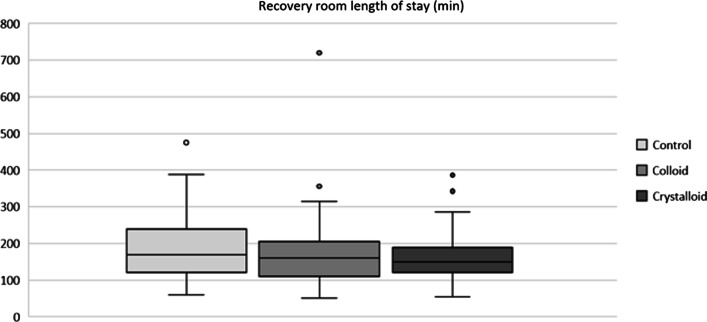
Total length of stay in the recovery room. The circles indicate outliers that lie outside the lower and upper quartiles

There was no significant difference in the rate of admission to PACU or ICU as well as the LOS in PACU and ICU (colloid group: median: 0 (range 0–1) d; crystalloid group: median: 0 (range 0–1) d; and controls: median: 0 (range 0–1) d). Length of stay in the recovery room was similar between all groups (Fig. 4). Patients in the colloid group had a shorter hospital length of stay compared to patients in the crystalloid group and control patients (colloid group: median: 9 (range 8–12) d; crystalloid group: median: 10 (range 9–13) d; and controls median: 11 (range 9–15) d).

## Discussion

Perioperative fluid management using a GDFT protocol with crystalloids in hip revision arthroplasty surgery was successfully implemented in daily clinical routine at a tertiary hospital. GDFT using crystalloids optimized SV without the need of an increased amount of volume to be administered compared to colloids. In addition, perioperative complications rates were reduced compared to a previous management without GDFT and comparable when using colloids. Interestingly, there may be a signal toward reduced hemorrhagic complications in the GFDT crystalloid group.

The concept of GDFT has been applied and used in several randomized controlled trials, as well as undergoing extensive meta-analyses, and seems to benefit patients with a high risk of perioperative mortality [23], as in the case of hip revision surgery [24]. It is associated with fewer postoperative complications, shorter hospital stays, reduction in postoperative complications as well as hospital length of stay [23, 25–29]. However, in previous studies, GDFT was mainly performed using colloid solutions, which are themselves associated with complications in certain patient populations. Considering the above-mentioned EMA-restriction for HES and several other studies, which showed severe side effects of colloids, crystalloids and 0.9% saline have been discussed as an alternative [30]. In the meantime, data exist demonstrating that GDFT using crystalloids may also be feasible in major surgery [20, 22, 31–33]. Therefore, the aim of this study was the implementation of a GDFT protocol using balanced crystalloids in hip revision surgery in daily clinical routine as part of a quality management project.

One significant concern applies to the overall amount of intravenous fluids needed for effective crystalloid based GDFT [33]. There are ambiguous results concerning the advantages and disadvantages of restrictive versus liberal fluid management. A restrictive fluid management strategy leads to faster recovery of pulmonary function and less postoperative complications [34–36]. In contrast, there is evidence that a liberal use of crystalloids during colorectal surgery leads to less postoperative nausea and vomiting (PONV) and pain [35]. The RELIEF-study, which included 3000 patients, also showed that restrictive volume management leads to higher rates of acute kidney dysfunction, renal replacement therapy and kidney transplantation [37]. In this context, an individualized, goal-directed fluid management to optimize SV and oxygen supply to the organs may be the most appropriate clinical approach, especially in high risk patients. As such, in our study, patients in the crystalloid and the colloid group, compared to the control group, received slightly more volume in total. The crystalloid group only received a non-significant greater amount of volume than the colloid group, in contrast to previous studies in which GDFT with colloids resulted in less total volume infusion [20, 22, 31–33]. A possible explanation for the higher doses of catecholamines in the crystalloid group might be the lesser intravasal volume effect of these agents compared to colloids. This might also explain the greater necessity of vasopressors in crystalloid group. However, all studies, including ours, were able to optimize SV by administering balanced crystalloids. Protocol adherence displays optimal performance of the attending anesthesiologist. A possible explanation for the difference between previous studies and our might be the additional use of inotropes compared to our controls with alteration of the Frank–Starling curve as the physiological basis as well as the timing of optimization.

Overall postoperative complications rates were similar between both GDFT patient groups and lower compared to control patients without GDFT in our study. This is in line with other studies confirming that postoperative complications can be mitigated by using a GDFT protocol for perioperative fluid management [12, 22, 32, 38–40]. The only complication difference within both GDFT groups was higher hemorrhagic complications within the colloid group, again confirming a previous study [31]. Finally, no differences in renal and arrhythmogenic complications between both intervention groups were found, which may also reflect statistical underpowering for these endpoints.

In relation to LOS, GDFT-based fluid management can shorten the length of hospital stay compared to fluid resuscitation without advanced hemodynamic monitoring or predefined goals [6, 12]. Previous findings have found that LOS for hospital, recovery room, PACU or ICU between patients receiving colloids or crystalloid-based GDFT do not significantly differ [18, 20, 22, 31, 33, 39, 41]. From these mentioned sources, our study confirms these findings in hip revision arthroplasty surgery. Most of the patients included in previous studies underwent abdominal or thoracic surgery and thus received postoperative treatment either at PACU or ICU. In our study, a significant number of enrolled patients were treated in the recovery room after surgery, highlighting an uneventful postoperative pathway without overt signs of clinically relevant fluid overload in the crystalloid group. Interestingly, the colloid group was associated with a shorter hospital length of stay compared to the crystalloid group by one day. It remains unclear if this is directly due to the usage of colloids or due to the diagnosis-related groups (DRGs), the reimbursement system for the inpatient sector in German hospitals. In addition, this study was not powered at all to evaluate such difference thoroughly.

The current study is limited by several factors. First of all, we compared patients who received crystalloid solutions to an ambispective cohort. This may skew the comparison as surgical techniques may have improved and therefore may have impacted the results of the study. Additionally, though “hip revision surgery” may present a homogenous cohort, we cannot exclude “surgical” differences (i.e., “easy” vs. “complex” revision) as we did not control for surgical case complexity. In addition, we did not differentiate between infected and aseptic revision procedures. We also collected data from during different time periods as opposed to simultaneous data collection. This may have led to an improvement in our internal SOPs as evidenced by the number of protocol deviations decreasing over time. Furthermore, this study was only single-centered. SV_trigger_ was determined during anesthesia and not prior to induction, which may have led to a false estimation of the true SV_trigger_. Also, during this time, patients were being positioned on the OR table, and therefore, movement artifacts during this process may have distorted the determination of the trigger value. Only one method of evaluating SV was examined. Exploring additional causes of hemodynamic beyond SV was not performed. Furthermore, the possible presence of existing or new perioperative heart failure was not taken into consideration. A recent Cochrane analysis underlined the usefulness of applying the concept of GDFT and stated that, at present, a distinct inotropic drug cannot be recommended [42]. An echocardiography is the only available hemodynamic monitoring device to fully evaluate the causes of hemodynamic instability in these complex patients. Therefore, future studies should evaluate GDFT concepts based on echocardiography in high-risk non-cardiac surgery patients. Another limitation was the fact that the application of colloids was also possible in the crystalloid group. This may have influenced the results. However, both groups received equal amounts of additional hemodynamic pharmacological support. Therefore, we assume a systematic error which may be negligible in clinical routine. Of note, in the Charité, 0.9% saline is not used anymore for perioperative and/or critically ill patients according to the corresponding Cochrane recommendation [43]. Another limitation lies in the calculation of the number of patients for the primary outcome parameter, as such we here can only present an explorative data analysis. This study was conceptualized as a “before-after comparison” within a quality management project at a large tertiary hospital in Germany. Thus, the results have to be understood as a successful implementation of a GDFT protocol based on crystalloids presented in an explorative character, which can only confirm previous evidence coming from RCTs. However, the translation of RCT evidence into daily clinical routine may be seen a strength of our study, hopefully encouraging others to also implement such a quality project. Due to the COVID-19 pandemic and its immense workload, the authors only have been able to compile their results within a manuscript somewhat delayed. However, still these results underline the necessity of well-organized local implementation projects.

## Conclusions

In conclusion, perioperative fluid management guided by a GDFT protocol using crystalloid solutions could be implemented in daily clinical routine and seems to be meaningful and viable. There was no difference found with regard to the increase in stroke volume and total volume received. Furthermore, the rate of perioperative complications was similar between GDFT crystalloid vs. colloid, with the crystalloid group having significantly less postoperative hemorrhagic complications. Finally, both GDFT groups showed significantly lowered rates of perioperative complications compared to the control group. Taken together, despite specific (side) effects of any kind of fluid, appropriate timing of fluid substitution within a GDFT protocol aiming at individually optimizing patients’ SV may be more important in order to improve patient’s outcome. Orthopedic surgeons should coordinate with their anesthesia team perioperatively to implement these findings within a jointly agreed standard operating procedure.

## Data Availability

The datasets generated and/or analyzed during the current study are not publicly available due to data protection issues but are available from the corresponding author upon reasonable request.

## Abbreviations

CI: Confidence interval
DRG: Diagnosis-related group
EMA: European Medicines Agency
GDFT: Goal-directed fluid therapy
ICU: Intensive care unit
LOS: Length of stay
MAP: Mean arterial pressure
PACU: Post-anesthesia care unit
PONV: Postoperative nausea and vomiting
RBC: Red blood cell
SOP: Standard operating procedure
SV: Stroke volume
